# Supplementary material for: Heat adaptation of phage T7 under an extended genetic code
Source: Virus Evol. 2021 Dec 1;7(2):veab100. doi: 10.1093/ve/veab100 (PMC8923235; doi:10.1093/ve/veab100)
Supplement: veab100_Supp [file veab100_supp.zip › Sequencing_Analysis.zip!/Sequencing_Analysis/S3/evidence/SNP_5.html]

BRESEQ :: Evidence


| Predicted mutation | | | | | | |
| --- | --- | --- | --- | --- | --- | --- |
| evidence | seq id | position | mutation | annotation | gene | description |
| RA | NC\_001604.1 | 20,544 | G→A | intergenic (–/–) | – / – | –/– |

| Read alignment evidence... | | | | | | | | | | | |
| --- | --- | --- | --- | --- | --- | --- | --- | --- | --- | --- | --- |
|  | seq id | position | | ref | new | freq | score (cons/poly) | reads | annotation | genes | product |
| \* | NC\_001604.1 | 20,544 | 0 | G | A | 81.5% | 2913.7 / inf | 1587 | intergenic (–/–) | *–/–* | –/– |
| Reads supporting (aligned to +/- strand):  **ref** base G (146/146);  **new** base A (608/680);  **total** (758/829) | | | | | | | | | | | |
| Fisher's exact test for biased strand distribution *p*-value = 3.99e-01 | | | | | | | | | | | |
| Kolmogorov-Smirnov test that lower quality scores support variant *p*-value = 1.00e+00 | | | | | | | | | | | |

Only 100 of 1591 total aligned reads displayed.  

`GAGAAACGAACAGGACTTGCGGAGGATGGCGCAAAGTCTGTCTATGAGCGTTTAAAGAACGACCGTGCTCCCTATGAGACACGCGCTCAGAATTGCGCTCAATATA‑CCATCCCATCATTGTTCCCTAAGGACTCCGATAACGCCTCTACAGATTATCAAACTCCGTGGCAAGCCGTGGGCGCTCGTGGTCTGAACAATCTAGCCTCTAAGCTCATGCTGGCTCTATTCCCTATGCAGACTTGGATGCGACTTACTATATCTGAATATGAAGCAAAGCAGTTACTGAGCGACCCCGATGGACTCGCTAAGGTCGATGAGGGCCTCTCGATGGTAGAGCGTATCATCATGAACTACATTGAGTCTAACAGTTACCGCGTGACTCTCTTTGAGGCTCTCAAACAGTTAGTCGTAGCTGGTAACGTCCTGCTGTACCTACCGGAACCGGAAGGGTCAAACTATAATCCCATGAAGCTGTACCGATTGTCTTCTTATGTGGTCCAACGAGACGCATTCGGCAACGTTCTGCAAATGGTGACTCGTGACCAGATAGCTTTTGGTGCTCTCCCTGAGGACATCCGTAAGGCTGTAGAAGGTCAAG  >  NC_001604.1/20246‑20843`  

                                                                                                                                                                                                                                                                                                           `|`                                                                                                                                                                                                                                                                                                             
`gagaAACGAACAGGACTTGCGGAGGATGGCGCAAAGTCTGTCTATGAGCGTTTAAAGAACGACCGTGCTCCCTATGAGACACGCGCTCAGAATTGCGCTCAATATA‑CCATCCCATCATTGTTCCCTAAGGACTCCGATAACGCCTCTACAGATTATCAAACTCCGTGGCAAGCCGTGGGCGCTCGTGGTCTGAACAATCTAGCCTCTAAGCTCATGCTGGCTCTATTCCCTATGCAGACTTGGATGCGACTTACTATATCTGAATATGAAGCAAAGCAGTTACTGAGCGACCCCGATGGAc                                                                                                                                                                                                                                                                                                           <  1:57860/301‑1 (MQ=255)`  
    `aaCGAACAGGACGTGCGGAGGATGGCGCAAAGTCTGTCTATGAGCGTTTAAAGAACGATCGTGCTCCCTATGAGACACGCGCTCAGAATTGCGCTCAATATACCCATCCCATCATTGTTCCCTAAGGACTCCGATAACGCCTCTACAGATTATCAAACTCCGTGGCAAGCCGTGGGCGCTCGTGGTCTGAACAATCTAGCCTCTAAGCTCATGCTGGCTCTATTCCCTATGCAGACTTGGATGCGACTTACTATATCTGAATATGAAGCAAAGCAGTTACTGAGCGACCCCGATGGACTCg                                                                                                                                                                                                                                                                                                        <  1:331410/301‑1 (MQ=255)`  
     `cggagatgtgtataagagacagggCGCAAAGTCTGTCTATGAGCGTTTAAAGAACGACCGTGCTCCCTATGAGACACGCGCTCAGAATTGCGCTCAATATA‑CCATCCCATCATTGTTCCCTAAGGACTCCGATAACGCCTCTACAGATTATCAAACTCCGTGGCAAGCCGTGGGCGCTCGTGGTCTGAACAATCTAGCCTCTAAGCTCATGCTGGCTCTATTCCCTATGCAGACTTGGATGCGACTTACTATATCTGAATATGAAGCAAAGCAGTTACTGAGCGACCCCGATGAACTCGCt                                                                                                                                                                                                                                                                                                      <  1:166841/279‑1 (MQ=255)`  
          `cAGGACTTGCGGAGGATGGAGCAAAGTCTGTCTATGAGCGTTTAAAGAACGACCGTGCTCCCTATGAGACACGCGCTCAGAATTGCGCTCAATATA‑CCATCCCATCATTGTTCCCTAAGGACTCAGATAACGCCTCTACAGATTATCAAACTCCGTGGCAAGCCGTGGGCGCTCGTGGTCTGAACAATCTAGCCTCTAAGCTCATGCTGGCTCTATTCCCTATGCAGACTTGGATGCGACTTACTATATCTGAATATGAAGCAAAGCAGTTACTGAGCGACCCCGATGGACTCGCTAAGGt                                                                                                                                                                                                                                                                                                 <  1:255670/301‑1 (MQ=255)`  
          `aaGGACTTGCGGAGGATGGCGCAAAGTCTGTCTATGAGCGTTTAAAGAACGACCGTGCTCCCTATGAGACACGCGCTCAGAATTGCGCTCAATATA‑CCATCCCATCATTGTTCCCTAAGGACTCCGATAACGCCTCTACAGATTATCAAACTCCGTGGCAAGCCGTGGGCGCTCGTGGTCTGAACAATCTAGCCTCTAAGCTCATGCTGGCTCTATTCCCTATGCAGACTTGGATGCGACTTACTATATCTGAATATGAAGCAAAGCAGTTACTGAGCGACCCCGATGGACTCGCTAAGGt                                                                                                                                                                                                                                                                                                 <  1:108390/300‑1 (MQ=255)`  
               `cTTGCGGAGGATGGCGCAAAGTCTGTCTATGAGCGTTTAAAGAACGACCGTGCTCCCTATGAAACACGCGCTCAGAATTGCGCTCAATATA‑CCATCCCATCATTGTTCCCTAAGGACTCCGATAACGCCTCTACAGATTATCAAACTCCGTGGCAAGCCGTGGGCGCTCGTGGTCTGAACAATCTAGCCTCTAAGCTCATGCTGGCTCTATTCCCTATGCAGACTTGGATGCGACTTACTATATCTGAATATGAAGCAAAGCAGTTACTGAGCGACCCCGATGAACTCGCTAAGGTCGATg                                                                                                                                                                                                                                                                                            <  1:308046/301‑1 (MQ=255)`  
                 `ggCGGAGGATGGCGCAAAGTCTGTCTATGAGCGTTTAAAGAACGACCGTGCTCCCTATGAGACACGCGCTCAGAATTGCGCTCAATATA‑CCATCCCATCATTGTTCCCTAAGGACTCCGATAACGCCTCTACAGATTATCAAACTCCGTGGCAAGCCGTGGGCGCTCGTGGTCTGAACAATCTAGCCTCTAAGCTCATGCTGGCTCTATTCCCTATGCAGACTTGGATGCGACTTACTATATCTGAATATGAAGCAAAGCAGTTACTGAGCGACCCCGATGAACTCGCTAAGGTCGATGAg                                                                                                                                                                                                                                                                                          <  1:230689/300‑1 (MQ=255)`  
                            `gcgcAAAGTCTGTCTATGAGCGTTTAAAGAACGACCGTGCTCCCTATGAGACACGCGCTCAGAATTGCGCTCAATATA‑CCATCCCATCATTGTTCCCTAAGGACTCCGATAACGCCTCTACAGATTATCAAACTCCGTGGCAAGCCGTGGGCGCTCGTGGTCTGAACAATCTAGCCTCTAAGCTCATGCTGGCTCTATTCCCTATGCAGACTTGGATGCGACTTACTATATCTGAATATGAAGCAAAGCAGTTACTGAGCGACCCCGATGAACTCGCTAAGGTCGATGAGGGCCTCTCGAt                                                                                                                                                                                                                                                                               <  1:336198/301‑1 (MQ=255)`  
                               `acagggctgtctATGAGCGTTTAAAGAACGACCGTGCTCCCTATGAGACACGCGCTCAGAATTGCGCTCAATATA‑CCATCCCATCATTGTTCCCTAAGGACTCCGATAACGCCTCTACAGATTATCAAACTCCGTGGCAAGCCGTGGGCGCTCGTGGTCTGAACAATCTAGCCTCTAAGCTCATGCTGGCTCTATTCCCTATGCAGACTTGGATGCGACTTACTATATCTGAATATGAAGCAAAGCAGTTACTGAGCGACCACGATGAACTCGCTAAGGTCGATGAGGGCCTCTCGATGGt                                                                                                                                                                                                                                                                            <  1:295941/295‑1 (MQ=255)`  
                                 `aaGTCTGTCTATGAGCGTTTAAAGAACGACCGTGCTCCCTATGAGACACGCGCTCAGAATTGCGCTCAATATA‑CCATCCCATCATTGTTCCCTAAGGACTCCGATAACGCCTCTACAGATTATCAAACTCCGTGGCAAGCCGTGGGCGCTCGTGGTCTGAACAATCTAGCCTCTAAGCTCATTCTGGCTCTATTCCCTATGCAGACTTGGATGCGACTTACTATATCTGAATATGAAGCAATGCAGTTACTGAGCGACCCCGATGAACTCCCTAAAGTCCATGAGGGCCTCTCGATGGTag                                                                                                                                                                                                                                                                          >  1:342427/1‑301 (MQ=255)`  
                                   `gtctgtctATGAGCGTTTAAAGAACGACCGTGCTCCCTATGAGACACGCGCTCAGAATTGCGCTCAATATA‑CCATCCCATCATTGTTCCCTAAGGACTCCGATAACGCCTCTACAGATTATCAAACTCCGTGGCAAGCCGTGGGCGCTCGTGGTCTGAACAATCTAGCCTCTAAGCTCATGCTGGCCCTATTCCCTATGCAGACTTGGATGCGACTTACTATATCTGAATATGAAGCAAAGCAGTTACTGAGCGACCCCGATGAACTCGCTAAGGTCGATGAGGGCCTCTCGATGGAagag                                                                                                                                                                                                                                                                        >  1:74366/1‑301 (MQ=255)`  
                                    `tctgtctATGAGCGTTTAAAGAACGACCGTGCTCCCTATGAGACACGCGCTCAGAATTGCGCTCAATATA‑CCATCCCATCATTGTTCCCTAAGGACTCCGATAACGCCTCTACAGATTATCAAACTCCGTGGCAAGCCGTGGGCGCTCGTGGTCTGAACAATCTAGCCTCTAAGCTCATGCTGGCTCTATTCCCTATGCAGACTTGGATGCGACTTACTATATCTGAATATGAAGCAAAGCAGTTACTGAGCGACCCCGATGAACTCGCTAAGGACGATGAGGGCCTCTCGATGGTAGAGc                                                                                                                                                                                                                                                                       <  1:188355/301‑1 (MQ=255)`  
                                     `atgtgtataagagacagaaGAACGACCGTGCTCCCTATGAGACACGCGCTCAGAATTGCGCTCAATATA‑CCATCCCATCATTGTTCCCTAAGGACTCCGATAACGCCTCTACAGATTATCAAACTCCGTGGCAAGCCGTGGGCGCTCGTGGTCTGAACAATCTAGCCTCTAAGCTCATGCTGGCTCTATTCCCTATGCAGACTTGGATGCGACTTACTATATCTGAATATGAAGCAAAGCAGTTACTGAGCGACCCCGATGAACTCGCTAAGGTCGATGAGGGCCTCTCGATGGTAGAGCg                                                                                                                                                                                                                                                                      <  1:136083/284‑1 (MQ=255)`  
                                           `aTGAGCGTCTAAAGAACCACCGTGCTCCCTATACGACACGCGCTCAGAATTGCGCTCAATATA‑CCATCCCATACTTTTTCCCTAAGGACTCCGATAACGCCTCTACAGATTATCAAACGCCGTGGCAAGCCTTGGGCGCTCGTGGTCTGAACAATCTAGCCTCTATGCTCATGCTGGCTCTATTCCCTATGCAGACTTGGATGCGACTTACTATATCTGAATATGAAGCAAAGCAGTTACTGAGCGACCCCGATGAACTCGCTAAGGTCGATGAGGGCCTCTCGATGGTAGAGCGTatcat                                                                                                                                                                                                                                                                <  1:45806/301‑1 (MQ=255)`  
                                                  `tttAACGAACGCCCGTGCTCCCGATGAGACACGCGCTCAGAATTTCGCTCAATATA‑CCATCCCATCATTGTTCCCTAAGGACTCCGATAACGCCTCTACAGATTATCAAACTCCGTGGCAAGCCGTGGGCGCTCGTGGTCTGAACAATCTAGCCTCTAAGCTCATGCTGGCTCTATTCCCTATGCAGACTTGGATGCGACTTACTATATCTGAATATGAAGCAAAGCAGTTACTGAGCGACCCCAATGAACTCGCTAAGGTCGATGAGGGCCTCTCGATGGTAGAGCGTATCATCATGAAc                                                                                                                                                                                                                                                         <  1:239480/301‑1 (MQ=255)`  
                                                      `aaGCACGACCGTGCTCCCTATGAGACACGCGCTCAGAATTGCGCTCAATATA‑CCATCCCATCATTGTTCCCTAAGGACTCCGATAACGCCTCTACAGATTATCAAACTCCGTGGCAAGCCGCGGGCGCTCGTGGTCTGAACAATCTAGCCTCTAAGCTCATGCTGGCTCTATTCCCTATGCAGACTTGGATGCGACTTACTATATCTGAATATGAAGCAAAGCAGTTACTGAGCGACCCCGATGAACTCGCTAAGGTCGATGAGGGCCTCTCGATGGTAGAGCGTATCATCATGAACTACa                                                                                                                                                                                                                                                     >  1:289359/1‑301 (MQ=255)`  
                                                      `aaGAACGACCGTGCTCCCTATGAGACACGCGCTCAGAATTGCGCTCAATATA‑CCATCCCATCATTGTTCCCTAAGGACTCCGATAACGCCTCTACAGATTATCAAACTCCGTGGCAAGCCGTGGGCGCTCGTGGTCTGAACAATCTAGCCTCTAAGCTCATGCTGGCTCTATTCCCTATGCAGACTTGGATGCGACTTACTATATCTGAATATTAAGCAAAGCAGTTACTGAGCGACCCCGATGAACTCGCTACGGTCGATGAGGGCCTCTCGATGGTAGAGctgtctcttatcaccatct                                                                                                                                                                                                                                                     >  1:155509/1‑283 (MQ=255)`  
                                                       `aGAACGACCGTGCTCCCTATGAGACACGCGCTCAGAATTGCGCTCAATATA‑CCATCCCATCATTGTTCCCTAAGGACTCCGATAACGCCTCTACAGATTATCAAACTCCGTGGCAAGCCGTGGGCGCTCGTGGTCTGAACAATCTAGCCTCTAAGCTCATGCTGGCTCTATTCCCTATGCAGACTTGGATGCGACTTACTATATCTGAATATGAAGCAAAGCAGTTACTGAGCGACCCCGATGAACTCGCTAAGGTCGATGAGGGCCTCTCGATGGTAGAGCGTATCATCATGAACTACAt                                                                                                                                                                                                                                                    <  1:79805/301‑1 (MQ=255)`  
                                                       `aGAACGACCGTGCTCCCTATGAGACACGCGCTCAGAATTGCGCTCAATATA‑CCATCCCATCATTGTTCCCAAAGGACTCCGATAACGCCTCTACAGATTATCAAACTCCGTGGCAAGCCGTGGGCGCTCGTGGTCTGAACAATCTAGCCTCTAAGCTCATGCTGGCTCTATTCCCTATGCAGACTTGGATGCGACTTACTATATCTGAATATGAAGCAAAGCAGTTACTGAGCGACCCCGATGAACTCGCTAAGGTCGATGAGGGCCTCTCGATGGTAGAGCGTATCATCATGAACTACAt                                                                                                                                                                                                                                                    <  1:196183/301‑1 (MQ=255)`  
                                                         `atcGACCGTGCTCCCTATGAGAATAGCACTCAGAATTACACTCAATATA‑CCATCCCATCAGTGTGCCCTAATGAATCCGATAACGCATCTACAGATGATCAAACTCCGTGGCAAGACGTGGGCGCTCGTGGTCGGAACAATCTATCCTCTAAGCTCATGCTGGCTCTATTCCCTATGCAGACTTGGATGCGACTTACTATATCTGAATATGAAGCAAAGCAGTTACTGAGCGACCCCGATGAACTCGCTAAGGTCGATGAGGGCCTCTCGATGGTAGAGCGTATCATCATGAACTACATTg                                                                                                                                                                                                                                                  <  1:257960/299‑1 (MQ=255)`  
                                                            `aagagacagcccTATGAGACACGCGCTCAGAATTGCGCTCAATATA‑CCATCCCATCATTGTTCCCTAAGGAATCCGATAACGCCTCTACAGATTATCAAACTCCGTGGCAAGCCGTGGGCGCTCGTGGTCTGAACAATCTAGCCTCTAAGCTCATGCTGGCTCTATTCCCTATGCAGACTTGGATGCGACTTACTATATCTGAATATGAAGCAAAGCAGTTACTGAGCGACCCCGATGAACTCGCTAAGGTCGATGAGGGCCTCTCGATGGTAGAGCGTATCATCATGAACTACATTGAGt                                                                                                                                                                                                                                               <  1:222332/292‑1 (MQ=255)`  
                                                                   `cTCCCTATGAGACACGCGCTCAGAATTGCGCTCAATATA‑CCATCCCATCATTGTTCCCTAAGGACTCCGATAACGCCTCTACAGATTATCAAACTCCGTGGCAAGCCGTGGGCGCTCGTGGTCTGAACAATCTAGCCTCTAAGCTCATGCTGGCTCTATTCCCTATGCAGACTTGGATGCGACTTACTATATCTGAATATGAAGCAAAGCAGTTACTGAGCGACCCCGATGGACTCGCTAAGGTCGATGAGGGCCTCTCGATGGTAGAGCGTATCATCATGAACTACATTGAGTCTAACAg                                                                                                                                                                                                                                        <  1:261064/301‑1 (MQ=255)`  
                                                                     `cccTATGAGACACGCGCTCAGAATTGCGCTCAATATA‑TCATCCCATCATTGTTCCCTAAGGACTCCGATAACGCCTCTACAGATTATCAAACTCCGTGGCAAGCCGTGGGCGCTCGTGGTCTGAACAATCTAGCCTCTAAGCTCATGCTGGCTCTATTCCCTATGCAGACTTGGATGCGACTTACTATATCTGAATATGAAGCAAAGCAGTTACTGAGCGACCCCGATGGACTCGCTAAGGTCGATGAGGGCCTCTCGATGGTAGAGCGTATCATCATGAACTACATTGAGTCTAAcctgt                                                                                                                                                                                                                                      >  1:209755/1‑297 (MQ=255)`  
                                                                     `cccTATGAGACACGCGCTCAGAATTGCGCTCAATATA‑CCATCCCATCATTGTTCCCTAAGGACTCCGATAACGCCTCTACAGATTATCAAACTCCGTGGCAAGCCGTGGGCGCTCGTGGTCTGAACAATCTAGCCTCTAAGCTCATGCTGGCTCTATTCCCTATGCAGACTTGGATGCGACTTACTATATCTGAATATGACGCAAAGCAGTTACTGAGCGACCCCGATGGACTCGCTAAGGTCGATGAGGGCCTCTCGATGGTAGAGCGTATCATCATGAACTACATTGAGTCTAACAGtt                                                                                                                                                                                                                                      >  1:111217/1‑301 (MQ=255)`  
                                                                     `cccTATGAGACACGCGCTCAGAATTGCGCTCAATATA‑CCATCCCATCATTGTTCCCTAAGGACTCCGATAACGCCTCTACAGATTATCAAACTCCGTGGCAAGCCGTGGGCGCTCGTGGTCTGAACAATCTAGCCTCTAAGCTCATGCTGGCTCTATTCCCTATGCAGACTTGGATGCGACTTACTATATCTGAATATGAAGCAAAGCAGTTGCTGAGCGACCCCGATGAACTCGCTAAGGTCGATGAGGGCCTCTCGATGGTAGAGCGTATCATCATGAACTACATTGAGTCTAAcctgt                                                                                                                                                                                                                                      >  1:72006/1‑297 (MQ=255)`  
                                                                       `cTATGAGACACGCGCTCAGAATTGCGCTCAATATA‑CCATCCCATCATTGTTCCCTAAGGACTCCGATAACGCCTCTACAGATTATCAAACTCCGTGGCAAGCCGTGGGCGCTCGTGGTCTGAACAATCTAGCCTCTAAGCTCATGCTGGCTCTATTCCCTATGAAGACTTGGATGCGCCTTACTATATCTGAATATGAAGCAAAGCAGTTACTGAGCGACCCCGATGGACTCGCTAAGGTCGATGAGGGCCTCTCGATGGTAGAGCGTATCATCCTGAACTACATTGAGTCTACCAGTTAc                                                                                                                                                                                                                                    >  1:100003/1‑301 (MQ=255)`  
                                                                       `cTATGAGACACGCGCTCAGAATTGCGCTCAATATA‑CCATCCCATCATTGTTCCCTAAGGACTCCGATAACGCCTCTACAGATTATCAAACTCCGTGGCAAGCCGTGGGCGATCGTGGTCTGAACAATCTAGCCTCTAAGCTCATGCTGGCTCTATTCCCTATGCAGACTTGGATGCGACTTACTATATCTGAATATGAAGCAAAGCAGTTACTGAGCGACCCCGATGAACTCGCTAAGGTCGATGAGGGCCTCTCGATGGTAGAGCGTATCATCATGAACTACATTGAGTCTAAcctgtct                                                                                                                                                                                                                                    >  1:249355/1‑295 (MQ=255)`  
                                                                       `aatgtgtataagagacagtaaTTGCGCTCAATATA‑CCATCCCATCATTGTTCCCTAAGGACTCCGATAACGCCTCTACAGATTATCAAACTCCGTGGCAAGCCGTGGGCGCTCGTGGTCTGAACAATCTATCCTCTAAGCTCATGCTGGCTCTATTCCCTATGCAGACTTGGATGCGACTTACTATATCTGAATATGAAGCAAAGCAGTTACTGAGCGACCCCGATGAACTCGCTAAGGTCGATGAGGGCCTCTCGATGGTAGAGCGTATCATCATGAACTACATTGAGTCTAACAGTTAc                                                                                                                                                                                                                                    <  1:54923/282‑1 (MQ=255)`  
                                                                                       `cAGAATTGCGCTCAATATA‑CCATCCCATCATTGTTCCATAAGGACTCCGATAACGCCTCTACAGATTATCAAACTCCGTGGCAAGCCGTGGGCGCTCGTGGTCTGAACAATCTAGCCTCTAAGCTCATGCTGGCTCTATTCCCTATGCAGACTTGGATGCGACTTACTATATCTGAATATGAAGCAAAGCAGTTACTGAGCGACCCCGATGAACTCGCTAAGGTCGATGAGGGCCTCTCGATGGTAGAGCGTATCATCATGAACTACATTGAGTcctgtctcttatacacatctccgagcc                                                                                                                                                                                                                    >  1:48075/1‑275 (MQ=255)`  
                                                                                         `gAATTGCGCTCAATATA‑CCATCCCATCATTGTTCCCTAAGGACTCCGATAACGCCTCTACAGATTATCAAACTCCGTGGCAAGCCGTGGGCGCTCGTGGTCTGAACAATCTAGCCTCTAAGCTCATGCTGGCTCTATTCCCTATGCAGACTTAGATGCGACTTACTCTATCTGAGTATGAAGCAAAGCAGTTACTGAGCGACCCCGATGAACTCGCTAAGGTCGATGATGGCCTCTCGATGGTAGAGCTTATCATCATTAACTACATTCAGTCTAACAGTTACCTCGTGACTGTCTTTTAg                                                                                                                                                                                                                  >  1:176379/1‑301 (MQ=255)`  
                                                                                          `agacaggcTCAATATA‑CCATCCCATCATTGTTCCCTAAGGACTCCGATAACGCCTCTACAGATTATCAAACTCCGTGGCAAGCCGTGGGCGCTCGTGGTCTGAACAATCTAGCCTCTAAGCTCATGCTGGCTCTATTCCCTATGCAGACTTGGATGCGACTTACTATATCTGAATATGAAGCAAAGCAGTTACTGAGCGACCCCGATGAACTCGCTAAGGTCGATGAGGGCCTCTCGATGGTAGAGCGTATCATCATGAACTACATTGAGTCTAACAGTTACCGCGTGACTCTCTTTGAgg                                                                                                                                                                                                                 <  1:202457/295‑1 (MQ=255)`  
                                                                                                 `cTCAATATA‑CCATCCCATCATTGTTCCCTAAGGACTCCGATAACGCCTCTACAGATTATCAAACTCCGTGGCAAGCCGTGGGCGCTCGTGGTCTGAACAATCTAGCCTCTAAGCTCATGCTGGCTCTATTCCCTATGCAGACTTGGATGCGACTTACTATATCTGAATATGAAGCAAAGCAGTTACTGAGCGACCCCGATCAACTCGCTAAGGTCGATGAGGGCCTCTCTATGGTAGAGCGTCTCATCATGAACTACCTTGAGTCTAACAGTTACCGCTTGACTCTCTTTGAGGCTCTCaa                                                                                                                                                                                                          >  1:164265/1‑301 (MQ=255)`  
                                                                                                   `cAATATA‑CCATCCCATCATTGTTCCCTAAGGACTCCGATAACGCCTCTACAGATTATCAAACTCCGTGGCAAGCCGTGGGCGCTCGTGGTCTGAACAATCTAGCCTCTAAGCTCATGCTGGCTCTATTCCCTATGCAGACTTGGATGCGACTTACTATATCTGAATATGAAGCAAAGCAGTTACTGCGCGACCCCGATTAACTCGCTAAGGTCGATGAGGGCCTCTCGATGGTAGAGCGTATCATCATGAACTACATTGCGTCTAACAGTTACCGCGTGACTCTCTTTGAGGCTCTCAAAc                                                                                                                                                                                                        >  1:151072/1‑301 (MQ=255)`  
                                                                                                   `cAATATA‑CCATCCCATCATTGTTCCCTAAGGACTCCGATAACGCCTCTACAGATTATCAAACTCCGTGGCAAGCCGTGGGCGCTCGTGGTCTGAACAATCTAGCCTCTAAGCTCATGCTGGCTCTATTCCCTATGCAGACTTGGATGCGACTTACTATATCTGAATATGAAGCAAAGCAGTTACTGAGCGACCCCGATGGACTCGCTAAGGTCGATGATGGCCTCTCGATGGTAGCGCGTATCCTCATGAACTACATTGAGTCTAACAGTTACCGCGTGACTCTCTTTGAGGCTCTCCAAc                                                                                                                                                                                                        >  1:104739/1‑301 (MQ=255)`  
                                                                                                   `cAATATA‑CCATCCCATCATTGTTCCCTAAGGACTCCGATAACGCCTCTACAGATTATCAAACTCCGTGGCAAGCCGTGGGCGCTCGTGGTCTGAACAATCTAGCCTCTAAGCTCATGCTGGCTCTATTCCCTATGCAGACTTGGATGCGACTTACTATATCTGAATATGAAGCAAAGCAGTTACTGAGCGACCCCGATGAACCCGCTAAGGTCGATGAGGGCCTCTCGATGGTAGAGCGTATCATCATGAACTACATTGAGTCTAACAGTTACAGCGTGACTCTCTTTGAGGCTCTCaaaa                                                                                                                                                                                                        >  1:340111/1‑300 (MQ=255)`  
                                                                                                   `cAATATA‑CCATCCCATCATTGTTCCCTAAGGACTCCGATAACGCCTCTACAGATTATCAAACTCCGTGGCAAGCCGTGGGCGCTCGTGGTCTGAACAATCTAGCCTCTAAGCTCATGCTGGCTCTATTCCCTATGCAGACTTGGATGCGACTTACTATATCTGAATATGAAGCAAAGCAGTTACTGAGCGAACCCGCTGAACTCGCTAAGGTCGATCAGGGCCTCTCGATGGTAGAGCGTATCCTCATGAACTACATTGAGTCTAACAGTTACCGCGTGACTCTCTTTGAGGCTCTTaaaa                                                                                                                                                                                                        >  1:130223/1‑300 (MQ=255)`  
                                                                                                   `aaaTATA‑CCAGCCCATCAGTGTTCCCTAAGGACTCCGATAACGCCTCTAACGATTATCAAACTCCGTGGCAAGCCGTGGGCGCTCGTGGTCTGAACAATCTAGCCTCTAAGCTCATGCTGGCTCTATTCCCTATGCAGACTTGGATGCGACTTACTATATCTGAATATGAAGCAAAGCAGTTACTGAGCGACCCCGATGAACTCGCTAAGGTCGATGAGGGCCTCTCGATGGTAGAGCGTATCATCATGAACTACATTGAGTCTAACAGTTACCGCGTGACTCTCTTTGAGGCTCTCAAAc                                                                                                                                                                                                        <  1:16163/300‑1 (MQ=255)`  
                                                                                                    `cggagatgtgtataagagacagtCCCTAAGGACTCCGATAACGCCTCTACAGATTATCAAACTCCGTGGCAAGCCGTGGGCGCTCGTGGTCTGAACAATCTAGCCTCTAAGCTCATGCTGGCTCTATTCCCTATGCAGACTTGGATGCGACTTACTATATCTGAATATGAAGCAAAGCAGTTACTGAGCGACCCCGATGAACTCGCTAAGGTCGATGAGGGCCTCTCGATGGTAGAGCGTATCATCATGAACTACATTGAGTCTAACAGTTACCGCGTGACTCTCTTTGAGGCTCTCAAAc                                                                                                                                                                                                        <  1:252015/279‑1 (MQ=255)`  
                                                                                                     `atatA‑CCATCCCATCATTGTTCCCTAAGGACTCCGATAACGCCTCTACAGATTATCAAACTCCGTGGCAAGCCGTGGGCGCTCGTGGTCTGAACAATCTAGCCTCTAAGCTCATGCTGGCTCTATTCCCTATGCAGACTTGGATGCGACTTACTATATCTGAATATGAAGCAAAGCAGTTACTGAGCGACCCCGATGACCTCGCTACGGTCGATAAGGGCCTCTCGATGGAAGAGCGTATCATCATGAACTACATTAAGTCTAACCTTTACCACGTGACTCTCTTTTAGGCTCTCCAACAg                                                                                                                                                                                                      >  1:286109/1‑301 (MQ=255)`  
                                                                                                      `ggagatgtgtataagagacatCCCTAAGGACTCCGATAACGCCTCTACAGATTATCAAACTCCGTGGCAAGCCGTGGGCGCTCGTGGTCTGAACAATCTAGCCTCTAAGCTCATGCTGGCTCTATTCCCTATGCAGACTTGGATGCGACTTACTATATCTGAATATGAAGCAAAGCAGTTACTGAGCGACCCCGATGAACTCGCTAAGGTCGATGAGGGCCTCTCGATGGTAGAGCGTATCATCATGAACTACATTGAGTCTAACAGTTACCGCGTGACTCTCTTTGAGGCTCTCAAACAg                                                                                                                                                                                                      <  1:90779/281‑1 (MQ=255)`  
                                                                                                       `ata‑CCATCCCATCATTGTTCCCTAAGGACTCCGATAACGCCTCTACAGATTATCAAACTCCGTGGCAAGCCGTGGGCGCTCGTGGTCTGAACAATCTAGCCTCTAAGCTCATGCTGGCTCTATTCCCTATGCAGACTTGGATGCGACTTACTATATCTGAATATGAAGCAAAGCAGTTACTGAGCGACCCCGATGAACTCGCTAAGGTCGATGAGGGCCTCTCGATGGTAGAGCGTATCATCATGAACTACATTGAGTCTAACAGTTACCGCGTGACTCTCTTTGAGGCTCTCAAACAGtt                                                                                                                                                                                                    <  1:345661/301‑1 (MQ=255)`  
                                                                                                             `aataaggaacagtccccTAAGAACTCCGATAACGGCTCGACAGATTATCAAACTCCGTGGCAAGCCGTGGGCGCTCGTGGTCTGAACAATCTAGCCTCTAAGCTCATGCTGGCTCTATTCCCTAGGCAGACTTGGATGCGACTTACTATATCTGAATATGAAGCAAAGCAGTTACTGAGCGACCCCGATGAACTCGCTAAGGTCGATGAGGGCCTCTCAATGGTAGAGCGTATCATCATGAACTACATTGAGTCTAACAGTTACCGCGTGACTCTCTTTGAGGCTCTCAAACAGTTAGTCg                                                                                                                                                                                               <  1:38080/287‑1 (MQ=255)`  
                                                                                                              `tcccatcATTGTTCCCTAAGGACTCCGATAACGCCTCTACAGATTATCTAACTCCGTGGCAAGCCGTGGGCGCTCGTGGTCCGAACATTCTCGCCTCTAAGCTCATGCTGCCTCTATTCCCCATGCATACTTGGATCCGACTTACTATATCTGAATATGAAGCAAAGCAGTTCCTGAGCCACCCCGATGAACTCGCTAAGGTCGATCAGGGCCTCTCCATGGTAGATCGTATCATCATGACCTCCATTGAGTCTAACAGTTACCGCGTTACTCTCTTTGCGGCTCTCAAACCGTTAGttgc                                                                                                                                                                                              >  1:9389/1‑298 (MQ=255)`  
                                                                                                                `ccatcATTGTTCCCTAAGGACTCCGATAACGCCTCTACAGATTATCAAACTCCGTGGCAAGCCGTGGGCGCTCGTGGTCTGAACAATCTAGCCTCTAAGCTCATGCTGGCTCTATTCCCTATGCAGACTTGGATGCGACTTACTATATCTGAATATGAAGCAAAGCAGTTACTGAGCGACCCCGATGGACTCGCTAAGGTCGATGAGGGCCTCTCGATGGTAGAGCGTATCATCATGAACTACATTGAGTCTAACAGTTACCGCGTGACTCTCTTTGAGGCTCTCAAACAGTTAGTcatgt                                                                                                                                                                                            >  1:9671/1‑297 (MQ=255)`  
                                                                                                                  `atcatTGTTCCCTAAGGACTCCGATAACGCCTCTACAGATTATCAAACTCCGTGGCAAGCCGTGGGCGCTCGTGGTCTGAACAATCTAGCCTCTAAGCTCATGCTGGCTCTATTCCCTATGCAGACTTGGATGCGACTTACTATATCTGAATATGAAGCAAAGCAGTTACTGAGCGACCCCGATGAACTCGCTAAGGTCGATGAGGGCCTCTCGATGGTAGAGCGTATCATCATGAACTACATTGAGTCTAACAGTTACCGCGTGACTCTCTTTGAGGCTCTCAAACAGTTAGTCGTAGCt                                                                                                                                                                                          <  1:179006/301‑1 (MQ=255)`  
                                                                                                                   `tcatTGTTCCCTACGGACTCCGATAACGCCTCTACAGATTATCAAACTCCGTGGCAAGCCGTGGGCGCTCGTGGTCTGAACAATCTAGCCTCTAAGCTCATGCTGGCTCAATTCCCTATGCAGACTTGGATGCGACTTACTATATCTGAATATGAAGCAAAGCAGTTACTGAGCGACCCCGATGAACTCGCTAAGGTCGATGAGGGCCTCTCGATGGTAGAGCGTATCATCATGAACTACATGGAGTCTAACAGTTACCGCGTGACTCCCTTTGAGGCTCTCAAACAGTTAGCCGTAGTTg                                                                                                                                                                                         >  1:234494/1‑301 (MQ=255)`  
                                                                                                                        `gTTCCCTAAGGACTCCGATAACGCCTCTACAGATTATCAAACTCCGTGGCAAGCCGTGGGCGCTCGTGGTCTGAACAATCTAGCCTCTAAGCTCATGCTTGCTCTATTCCCTATGCAGACTTGGATGCGACTTACTATATCTGAATATGAAGCAAAGCAGTTACTGAGCGCCCCCGATGAACTCGCTAAGGTCGATGAGGGCCTCTCGATGGTAGAGCGTATCATCATGAACTACATTGAGTCTAACAGTTACCGCGTGACTCTCTTTGAGGCTCTCAAACAgctgtctcttatacacact                                                                                                                                                                                    >  1:43012/1‑283 (MQ=255)`  
                                                                                                                                 `ggACTTCGATAACGCCTCTACAGATTATCAAACTCCGTGGCAAGCCGTGGGCGCTCGTGGTCTGAACAATCTAGCCTCTAAGCTCATGCTGGCTCTATTCCCTATGCAGACTTGGATGCGACTTACTATATCTGAATATGAAGCAAAGCAGTTACTGAGCGACCCCGATGAACTCGCTAAGGTCGATGAGGGCCTCTCGATGGTAGAGCGTATCATCATGAACTACATTGAGTCTAACAGTTACCGCGTGACTCTCTTTGAGGCTCTCAAACAGTTAGCAGTAACTGGTAACGTCatgctg                                                                                                                                                                           >  1:182652/1‑301 (MQ=255)`  
                                                                                                                                 `ggACTCCGATAACGCCTCTACCGTTTATCAAACTCCGTGGCAAGCCGTGGGCGCTCGTGGTCTGAACAATCTAGCCTCTAAGCTCATGCTGGCTCTATTCCCTATGCAGACTTGGATGCGACTTACTATATCTGAATATGAAGCAAAGCAGTTACTGAGCGACCCCGATGGACTCGCTAAGGTCGATGAGGGCCTCTCGATGGTAGAGCGTATCATCATGAACTACATTGAGTCTAACAGTTACCGCGTGACTCTCTTTGAGGCTCTCAAACAGTTAGTCGTAGCTGGTAACGTCctgctg                                                                                                                                                                           <  1:192938/301‑1 (MQ=255)`  
                                                                                                                                 `ggACTCCGATAACGCCTCTACAGATTATCAAACTCCGTGGCAAGCCGTGGGCGCTCGTGGTCTGAACAATCTAGCCTCTAAGCTCATGCTGGCTCTATTCCCTATGCAGACTTGGATGCGACTTACTATATCTGAATATGAAGCAAAGCAGTTACTGAGCGACCCCGATGGACTCGCTAAGGTCGATGAGGGCCTCTCGATGGTAGAGCGTATCATCATGAACTACATTGAGTCTAACAGTTACCGCGTGACTCTCTTTGAGGCTCTCAAACAGTTAGTCGTAGCTGGTAACGTCctgctg                                                                                                                                                                           >  1:35197/1‑301 (MQ=255)`  
                                                                                                                                    `tcggagatgtgtataagagacagaTCAAACTCCGTGGCAAGCCGTGGGCGCTCGTGGTCTGAACAATCTAGCCTCTAAGCTCATGCTGGCTCTATTCCCTATGCAGACTTGGATGCGACTTACTATATCTGAATATGAAGCAAAGCAGTTACTGAGCGACCCCGATGAACTCGCTAAGGTCGATGAGGGCCTCTCGATGGTAGAGCGTATCATCATGAACTACATTGAGTCTAACAGTTACCGCGTGACTCTCTTTGAGGCTCTCAAACAGTTAGTCGTAGCTGGTAACGTCCTGCTGtac                                                                                                                                                                        <  1:76913/278‑1 (MQ=255)`  
                                                                                                                                    `cTCCGATAACGCCTCTACAGATTATCAAACTCCGTGGCAAGCCGTGGGCGCTCGTGGTCTGAACCATCTAGCCTCTAAGCTCATGCTGGCTCTATTCCCTATGCAGACTTGGATGCGACTGACTATATCTGAATATGAAGCAAAGCAGTTACTGAGCGACCCCGATGAACTCGCTAAGGTCGATGAGGGCCTCTCGATGGTAGAGCGTATCATCATGAACTACATTGAGTCTAACAGTTACCGCGTGACTCTCTTTGAGGCTCTCAAACAGTTAGTCGTAGCTGGTAACGTCCTGCTGtac                                                                                                                                                                        <  1:133097/301‑1 (MQ=255)`  
                                                                                                                                    `cTCCGATAACGCCTCTACAGATTATCAAACTCCGTGGCAAGCCGTGGGCGCTCGTGGTCTGAACAATCTAGCCTCTAAGCTCATGCTGGCTCTATTCCCTATGCAGACTTGGATGCGACTTACTATATCTGAATATGAAGCAAAGCAGTTACTGAGCGACCCCGATGAACTCGCTAAGGTCGATGAGGGCCTCTCGATGGTAGAGCGTATCATCATGAACTACATTGAGTCTAACAGTTACCGCGTGACTCCCTTTGAGGCTCTCAAACAGTTAGTCGTAGCTGGTAACGTCCTGCTGtac                                                                                                                                                                        >  1:300751/1‑301 (MQ=255)`  
                                                                                                                                    `cTCCGATAACGCCTCGACAGATTATCAAACTCCGTGGCAAGCCGTGGGCGCTCGTGGTCTGAACAATCTAGCCTCTAAGCTCATGCTGGCTCTATTCCCTATGCAGACTTGGATGCGACTTACTATATCTGAATAGGAAGCAAAGCAGTTACTGAGCGACCCCGATGAACTCGCTAAGGTCGATGAGGGCCTCTCGATGGTAGAGCGTATCATCATGAACTACATTGAGTCTAACAGTTACCGCGTGACTCTCTGTGAGGCTCTCAAACAGTTAGTCGTAGCTGGTAACGTCCTGCTGtac                                                                                                                                                                        <  1:28261/301‑1 (MQ=255)`  
                                                                                                                                         `aTAACGCCTCTACAGATTATCAAACTCCGTGGCAAGCCGTGGGCGCTCGTGGTCTGAACAATCTAGCCTCTAAGCTCATGCTGGCTCTATTCCCTATGCAGACTTGGATGCGACTTACTATATCTGAATATGAAGCAAAGCAGTTACTGAGCGACCCCGATGAACTCGCTAAGGTCGATGAGGGCCTCTCGATGGTAGAGCGTATCATCATGAACTACATTGAGTCTAACAGTTACCGCGTGACTCTCTTTGAGGCTCTCAAACAGTTAGTCGTAGCTGGTAACGTCCTGCTGtacctacc                                                                                                                                                                   <  1:39680/301‑1 (MQ=255)`  
                                                                                                                                         `aTAACGCCTCTACAGATTATCAAACTCCGTGGCAAGCCGTGGGCGCTCGTGGTCTGAACAATCTAGCCTCTAAGCTCATGCTGGCTCTATTCCCTATGCAGACTTGGATGCGACTTACTATATCTGAATATGAAGCAAAGCAGTTACTGAGCGACCCCGATGAACTCGATAAGGTCGATGAGGGCCTCTCGATGGTAGAGCGTATCATCATGAACTACATTGAGTCTAACAGTTACCGCGTGACTCTCTTTGAGGCTCTCAAACAGTTAGTCGTAGCTGGTAACGTCCTGCTGtacctacc                                                                                                                                                                   <  1:87392/301‑1 (MQ=255)`  
                                                                                                                                                  `ctACAGATTATCAAACTCCGTGGCAAGCCGTGGGCGCTCGTGGTCTGAACAATCTAGCCTCTAAGCTCATGCTGGCTCTATTCCCTATGCAGACTTGGATGCGACTTACTATATCTGAATATGAAGCAAAGCAGTTACTGAGCGACCCCGATGAACTCGCTAAGGTCGATGAGGGCCTCTCGATGGTAGAGCGTATCATCATGAACTACATTGAGTCTAACAGTTACCGCGTGACTCTCTTTGAGGCTCTCAAACAGTTAGTCGTAGCTGGTAACGTCctgctgctgtctcttatacacat                                                                                                                                                          >  1:328283/1‑284 (MQ=255)`  
                                                                                                                                                       `gATTATCAAACTCCGTGGCAAGCCGTGGGCGCTCGTGGTCTGAACAATCTAGCCTCTAAGCTCATGCTGGCTCTATTCCCTATGCAGACTTGGATGCGCCTTACTATATCTGAATATGAAGCAAAGCAGTTACTGAGCGACCCCGATGAACTCGCTAAGGTCGATGAGGGCCTCTCGATGGTAGAGCGTATCATCATGAACTACATTGAGTCTAACAGTTACCGCGTGACTCTCTTTGAGGCTCTCAAACAGTTAGTCGTAGCTGGTAACGTCCTGCTGTACCTACCGGAACCGGCAGGGt                                                                                                                                                     >  1:19854/1‑301 (MQ=255)`  
                                                                                                                                                         `ttATCAAACTCCGTGGCAAGCCGTGGGCGCTCGTGGTCTGAACAATCTAGCCTCTAAGCTCATGCTGGCTCTATTCCCTATGCAGACTTGGATGCGACTTACTATATCTGAATATGAAGCAAAGCAGTTACTGAGCGACCCCGATGAACTCGCTAAGGTCGATGAGGGCCTCTCGATGGTAGAGCGTATCATCATGAACTACATTGAGTCTAACAGTTACCGCGTGACTCTCTTTGAGGCTCTCAAACAGTTAGTCGTAGCTGGTAACGTCCTGCTGTACCTACCGGAACCGGAAGGGTCa                                                                                                                                                   >  1:242733/1‑301 (MQ=255)`  
                                                                                                                                                         `ttATCAAACTCCGTGGCAAGCCGTGGGCGCTCGTGGTCTGAACAATCTAGCCTCTAAGCTCATGCTGGCTCTATTCCCTATGCAGACTTGGATGCGACTTACTATATCTGAATATGAAGCAAAGCAGTTACTGAGCGACCCCGATGAACTCGCTAAGGTCGATGAGGGCCTCTCGATGGTAGAGCGTATCATCATGAACTACATTGAGTCTAACAGTTACCGCGTGACTCTCTTTGAGGCTCTCAAACAGTTAGTCGTAGCTGGTAACGTCCTGCTGTACCTACCGGAACAGGAAGGGTCa                                                                                                                                                   >  1:52344/1‑301 (MQ=255)`  
                                                                                                                                                             `cAAACTCCGTGGCAAGCCGTGGGCGCTCGTGGTCTGAACAATCTAGCCTCTAAGCTCATGCTGGCTCTATTCCCTATGCAGACTTGGATGCGACTTACTATATCTGAATATGAAGCAAAGCAGTTACTGAGCGACCCCGATGAACTCGCTAAGGTCGATGAGGGCCTCTCGATGGTAGAGCGTATCATCATGAACTACATTGAGTCTAACAGTTACCGCGTGACTCTCTTTGAGGCTCTCAAACAGTTAGTCGTAGCTGGTAACGTCCTGCTGTACCTACCGGAACCGGAAGGGTCAAACt                                                                                                                                               <  1:305235/301‑1 (MQ=255)`  
                                                                                                                                                                  `cccGTGGCAAGCCGTGGGCGCTCGTGGTCTGAACAATCTAGCCTCTAAGCTCATGCTGGCTCTATTCCCTATGCAGACTTGGATGCGACTTACTATATCTGAATATGAAGCAAAGCAGTTACTGAGCGACCCCGATGAACTCGCTAAGGTCGATGAGGGCCTCTCGATGGTAGAGCGTATCATCATGAACTACATTGAGTCTAACAGTTACCGCGTGACTCTCTTTGAGGCTCTCAAACAGTTAGTCGTAGCTGGTAACGTCCTGCTGTACCTACCGGAACCGGAAGGGTCAAACTATAAt                                                                                                                                          <  1:174263/300‑1 (MQ=255)`  
                                                                                                                                                                    `cGTGGCAAGCCGTGGGCGCTCGTGGTCTGAACAATCTAGCCTCTAAGCTAATGCTGGCTCTATTCCCTATGCAGACTTGGATGCGACTTACTATATCTGAATATGAAGCAAAGCAGTTACTGAGCGACCCCGATGAACTCGCTAAGGTCGATGAGGGCCTCTCGATGGTAGAGCGTATCATCATGAACTACATTGAGTCTAACAGTTACCGCGTGACTCTCTTTGAGGCTCTCAAACAGTTAGTCGTAGCTGGTAACGTCCTGCTGTACCCACCGGAACCGGAAGGGTCAAACTATAATcc                                                                                                                                        <  1:116600/301‑1 (MQ=255)`  
                                                                                                                                                                    `cGTGGCAAGCCGTGGGCGCTCGTGGTCTGAAAAATCTAGCCTCTAAGCTCATGCTGGCTCTATTCCCTATGAAGACTTGGATGCGACTTACTATATCTGAATATGAAGCAAAGCAGTTACTGAGCGACCCCGATGGACTCGCTAAGGTCGATGAGGGCCTCCCGATGGTAGAGCGTATCATCATGAACTACATTGAGTCTAACAGTTACCGCGTGACTCTCTTTGAGGCTCTCAAACAGTTAGTCGTAGCTGGTAACGTCCTGCTGTACCTACCGGAACCGGAAGGGTCAAACTATAATcc                                                                                                                                        <  1:185713/301‑1 (MQ=255)`  
                                                                                                                                                                                 `gggCGCTCGTGGTCTGAACAATCTAGCCTCTAAGCTCATGCTGGCTCTATTCCCTATGCAGACTTGGATGCGACTTACTATATCTGAATATGAAGCAAAGCAGTTACTGAGCGACCCCGATGAACTCGCTAAGGTCGATGAGGGCCTCTCGATGGTAGAGCGTATCATCATGAACTACATTGAGTCTAACAGTTACCGCGTGACTCTCTTTGAGGCTCTCAAACAGTTAGTCGTAGCTGGTAACGTCCTGCTGTACCTACCGGAACCGGAAGGGTCAAACTATAATCCCATGAAGCTGTAc                                                                                                                           <  1:215240/301‑1 (MQ=255)`  
                                                                                                                                                                                   `cctcTCGTGGTCTGAACAATCTAGCCTCTAAGCTCATGCTGGCTCTATTCCCTATGCAGACTTGGATGCGACTTACTATATCTGAATATGAAGCAAAGCAGTTACTGAGCGACCCCGATGAACTCGCTAAGGTCGATGAGGGCCTCTCGATGGTAGAGCGTATCATCATGAACTACATTGAGTCTAACAGTTACCGCGTGACTCTCTTTGAGGCTCTCAAACAGTTAGTCGTAGCTGGTAACGTCCTGCTGTACCTACCGGAACCGGAAGGGTCAAACTATAATCCCATGAAGCTGTACCg                                                                                                                         <  1:158118/298‑1 (MQ=255)`  
                                                                                                                                                                                      `cTCGTGGTCTGAACAATCTAGCCTCTAAGCTCATGCTGGCTCTATTCCCTATGCAGACTTGGATGCGACTTACTATATCTGAATATGAAGCAAAGCAGTTACTGAGCGACCCCGATGAACTCGCTAAGGTCGATGAGAGCCTCTCGATGGTAGAGCGTATCATCATGAACTACATTGAGTCTAACAGTTACCGCGTGACTCTCTTTGAGGCTCTCAAACAGTTAGTCGTAGCTGGTAACGTCCTGCTGTACCTACCGGAACCGGAAGGGTCAAACTATCATCCAATGAAGCTGTAcctgtc                                                                                                                      >  1:144942/1‑297 (MQ=255)`  
                                                                                                                                                                                           `ggTCTGAACAATCTAGCCTCTAAGCTCATGCTGGCTCTATTCCCTATGCAGACTTGGATGCGACTTACTATATCTGAATATGAAGCAAAGCAGTTACTGAGCGACCCCGATGGACTCGCTAAGGTCGATGAGGGCCTCTCGATGGTAGAGCGTATCATCATGAACTACATTGAGTCTAACAGTTACCGCGTGACTCTCTTTGAGGCTCTCAAACAGTTAGTCGTAGCTGGTAACGTCCTGCTGTACCTCCCGGAACCGGAAGGGTCAATCTATAATCCCATGAAGcccttctctaatacac                                                                                                                 >  1:141539/1‑286 (MQ=255)`  
                                                                                                                                                                                                           `ccTCTAAGCTCATGCTGGCTCTATTCCCTATGCAGACTTGGATGCGACTTACTATATCTGAATATGAAGCAAAGCAGTTACTGAGCGACCCCGATGGACTCGCTAAGGTCGATGAGGGCCTCTCGATGGTAGAGCGTATCATCATGAACTACATTGAGTCTAACAGTTACCGCGTGACTCTCTTTGAGGCTCTCAAACAGTTAGTCGTAGCTGGTAACGTCCTGCTGTACCTACCGGAACCGGAAGGGTCAAACTATAATCCCATGAAGCTGTACCGATTGTCTTCTTATGTGGTCCAACg                                                                                                 <  1:303612/301‑1 (MQ=255)`  
                                                                                                                                                                                                           `ccTCTAAGCTCATGCTGGCTCTATTCCCTATGCAGACTTGGATGCGACTTACTATATCTGAATATGAAGCAAAGCAGTTACTGAGCGACCCCGATGAACTCGCTAAGGTCGATGAGGGCCTCTCGATGGTGGAGCGTATCATCATGAACTACATTGAGTCTAACAGTTACCGCGTGACTCTCTTTGAGGCTCTCAAACAGTTAGTCGTAGCTGGTAACGTCCTGCTGTACCTACCGGAACCGGAAGGGTCAAACTATAATCCCATGAAGCTGTACCGATTGTCTTCTTATGTGGTCCAACg                                                                                                 >  1:68006/1‑301 (MQ=255)`  
                                                                                                                                                                                                            `ctctAAGCTCATGCTGGCTCTATTCCCTATGCAGACTTGGATGCGACTTACTATATCTGAATATGAAGCAAAGCAGTTACTGAGCGACCCCGATGAACTCGCTAAGGTCGATGAGGGCCTCTCGATGGTAGAGCGTATCATCATGAACTACATTGAGTCTAACAGTTACCGCGTGACTCTCTTTGAGGCTCTCAAACAGTTAGTCGTAGCTGGTAACGTCCTGCTGTACCTACCGGAACCGGAAGGGTCAAACTATAATCCCATGAAGCTGTACCGATTGTCTTCTTATGTGGTCCAACga                                                                                                <  1:82295/301‑1 (MQ=255)`  
                                                                                                                                                                                                                `aaGCTCATGCTGGCTCTATTCCCTATGCAGACTTGGATGCGACTTACTATATCTGAATATGAAGCAAAGCAGTTACTGAGCGACCCCGATGAACTCGCTAAGGTCGATGAGGGCCTCTCGATGGTAGAGCGTATCATCATGAACTACATTGAGTCTAACAGTTACCGCGTGACTCTCTTTGAGGCTCTCAAACAGTTAGTCGTAGCTGGTAACGTCCTGCTGTACCTACCGGAACCGGAAGGGTCAAACTATAATCCCATGAAGCTGTACCGATTGTCTTCTTATGTGGTCCAACGAGACg                                                                                            <  1:280155/301‑1 (MQ=255)`  
                                                                                                                                                                                                                              `tctATTCCCTATGCAGACTTGGATGCGACTTACTATATCTGAATATGAAGCAAAGCAGTTACTGAGCGACCCCGATGAACTCGCTAAGGTCGATGAGGGCCTCTCGATGGTAGAGCGTATCATCATGAACTACATTGAGTCTAACAGTTACCGCGTGACTCTCTTTGAGGCTCTCAAACAGTTAGTCGTAGCTGGTAACGTCCTGCTGTACCTACCGGAACCGGAAGGGTCAAACTATAATCCCATGAAGCTGTACCGATTGTCTTCTTATGTGGTCCAACGAGACGCATTCGGCAACGtt                                                                              >  1:293782/1‑301 (MQ=255)`  
                                                                                                                                                                                                                               `ctATTCCCTATGCAGACTTGGATGCGACTTACTATATCTGAATATGACGCAAAGCAGTTACTGAGCGACCCCGATGAACTCGCTAAGGTCGATGAGGGCCTCTCGATGGTAGAGCGTATCATCATGAACTACATTGAGTCTAACAGTTACCGCGTGACTCTCTTTGAGGCTCTCAAACAGTTAGTCGTAACTGGTAACGTCCTGCTGTACCTACTGGACCCGGAATGGTCAAACTATAATCCCATGATTCTGTACCGATTGTCTTCTTATGTGGTCCAACGAGAAGCATTAGGCaaagctt                                                                             >  1:323008/1‑296 (MQ=255)`  
                                                                                                                                                                                                                                          `aagatgtatataagaaacagcTATATCTGAATATGAAGCAAAGCAGTTACTGAGCGACCCCGATGAACTCGCTAAGGTCGATGAGGGCCTCTCGATGGTAGAGCGTATCATCATGAACTACATTGAGTCTAACAGTTACCGCGTGACTCTCTTTGAGGCTCTCAAACAGTTAGTCGTAGCTGGTAACGTCCTGCTGTACCTACCGGAACCGGAAGGGTCAAACTATAATCCCATGAAGCTGTACCGATTGTCTTCTTATGTGGTCCAACGAGACGCATTCGGCAACGTTCTGCAAATGGTg                                                                  <  1:267064/281‑1 (MQ=255)`  
                                                                                                                                                                                                                                             `gACTTGGATGCGACTTACTATATCTGAATATGAAGCAAAGCAGTTACTGAGCGACCCCGATGAACTCGCTAAGGTCGATGAGGGCCTCTCGATGGTAGAGCGTATCATCATGAACTACATTGAGTCTAACAGTTACCGCGTGACTCTCTTTGAGGCTCTCAAACAGTTAGTCGTAGCTGGTAACGTCCTGCTGTACCTACCGGAACCGGAAGGGTCAAACTATAATCCCATGAAGCTGTACCGATTGTCTTCTTATGTGGTCCAACGAGACGCAtctgtctcttatacacatctccgagcc                                                               >  1:120076/1‑275 (MQ=255)`  
                                                                                                                                                                                                                                                        `gACTTCCTATATCTGAATATGAAGCAAAGCAGTTACTGAGCGACCCAGATGGACTCGCTAAGGTCGATGCGGGCCTCTCGATGGTAGAGCGTATCATCATGAACTACATTGAGTCTAACAGTTACCGCGTGACTCTCTTTGAGGCTCTCAAACAGTTAGTCGTAGCTGGTAACGTCCTGCTGTACCTACCGGAACCGGAAGGGTCAAACTATAATCCCATGAAGCTGTACCGATTGTCTTCTTATGTGGTCCAACGAGACGCATTCGGCAACGTTCTGCAAATGGCGACTCGTGACCAGAt                                                    <  1:246718/301‑1 (MQ=255)`  
                                                                                                                                                                                                                                                        `gACTTACTATATCTGAATATGAAGCAAAGCAGTTACTGAGCGACCCCGATGAACTCGCTAAGGTCGATGAGGGCCTCTCGATGGTAGAGCGTATCATCATGAACTACATTGAGTCTAACAGTTACCGCGTGACTCTCTTTGAGGCTCTCAAACAGTTAGTCGTAGCTGGTAACGTCCTGCTGTACCTACCGGAACCGGAAGGGTCAAACTATAATCCCATGAAGCTGTACCGCTTGTCTTCTTATGTGGTCCAACGAGACGCATTCGGCAACGTTCTGCAAATGGTGACTCGTGACCAGAt                                                    >  1:313085/1‑301 (MQ=255)`  
                                                                                                                                                                                                                                                            `tACTATATCTGAATATGAAGCAAAGCAGTTACTGAGCGACCCCGATGAACTCGCTAAGGTCGATGAGGGCCTCTCGATGGTAGAGCGTATCATCATGAACTACATTGAGTCTAACAGTTACCGCGTGACTCTCTTTGAGTCTCTCAAACAGTTAGTCGTAGCTGGTAACGTCCTGCTGTACCTACCGGAACCGGACGGGTCAAACTATAATCCCATGAAGCTGTACCGATTGTATTCTTATGTGGTCCAACGAGACGAATTAGGCAACGTTCTGCCAATGGTGACTCGTTCCCATATAGCt                                                >  1:20557/1‑301 (MQ=255)`  
                                                                                                                                                                                                                                                            `tACTATATCTGAATATGAAGCAAAGCAGTTACTGAGCGACCCCGATGAACTCGCTAAGGTCGATGAGGGCCTCTCGATGGTAGAGCGTATCATCATGAACTACATTGAGTCTAACAGTTACCGCGTGACTCTCTTTGAGGCTCTCAAACAGTTAGTCGTAGCTGGTAACGTCCTGCTGTACCTACCGGAACCGGAAGGGTCAAACTATAATCCCATGAAGCTGTTCCGATTGTCTTCTTATGTGGTCCAACGAGACGCATTCGGCAACGTTCTGCAAATGGTGACTCGTGAcctgtctctt                                                >  1:263992/1‑293 (MQ=255)`  
                                                                                                                                                                                                                                                                        `atatGAAGCAAAGCAGTTACTGAGCGACCCCGATGAACTCGCTAAGGTCGATGAGGGCCTCTCGATGGTAGAGCGTATCATCATGAACTACATTGAGTCTAACAGTTACCGCGTGACTCTCTTTGAGGCTCTCAAACAGTTAGTCGTAGCTGGTAACGTCCTGCTGTACCTACCGGAACCGGAAGGGTCAAACTATAATCCCATGAAGCTGTACCGATTGTCTTCTTATGTGGTCCAACGAGACGCATTCGGCCACGTTCTGCAAATGGTGACTCGTGACCAGATAGCTTTTGGTGCtctc                                    >  1:325902/1‑301 (MQ=255)`  
                                                                                                                                                                                                                                                                        `atatGAAGCAAAGCAGTTACTGAGCGACCCCGATGAACTCGCTAAGGTCGATGAGGGCCTCTCGATGGTAGAGCGTATCATCATGAACTACATTGAGTCTAACAGTTACCGCGTGACTCTCTTTGAGGCTCTCAAACAGTTAGTCGTAGCTGGTAACGTCCTGCTGTACCTACCGGAACCGGAAGGGTCAAACTATAATCCCATGAAGCTGTACCGATTGTCTTCTTATGTGGTCCAACGAGACGCATTCGGCAACGTTCTGCAAATGGTGACTCGTGACCAGATAGCTTTTGGTGCtctc                                    >  1:212452/1‑301 (MQ=255)`  
                                                                                                                                                                                                                                                                             `aagcaaagcaGTTACTGAGCGACCCCGATGAACTCGCTAAGGTCGATGAGGGCCTCTCGATGGTAGAGCGTATCATCATGAACTACATTGAGTCTAACAGTTACCGCGTGACTCTCTTTGAGGCTCTCAAACAGTTAGTCGTAGCTGGTAACGTCCTGCTGTACCTACCGGAACCGGAAGGGTCAAACTATAATCCCATGAAGCTGTACCGATTGTCTTCTTATGTGGTCCAACGAGACGCATTCGGCAACGTTCTGCAAATGGTGACTCGTGACCAGATAGCtgtctcttatacacatct                               >  1:123483/1‑284 (MQ=255)`  
                                                                                                                                                                                                                                                                             `aagcaaagcaGTTACTGAGCGACCCCGATGAACTCGCTAAGGTCGATGAGGGCCTCTCGATGGTAGAGCGTATCATCATGAACTACATTGAGTCTAACAGTTACCGCGTGACTCTCTTTGAGGCTCTCAAACAGTTAGTCGTAGCTGGTAACGTCCTGCTGTACCTACCGGAACCGGAAGGGTCAAACTATAATCCCATGAAGCTGTACCGATTGTCTTCTTATGTGGTCCAACGAGACGCATTCGGCAACGTTCTGCAAATGGAGACTCGTGACCAGATAGCTTTTGGTGCTCTCCCTGa                               >  1:270422/1‑301 (MQ=255)`  
                                                                                                                                                                                                                                                                               `acaaagcaGTTACTGAGCGACCCCGATGAACTCGCTAAGGTCGATGAGGGCCTCTCGATGGTAGAGCGTATCATCATGAACTACATTGAGTCTAACAGTTACCGCGTGACTCTCTTTGAGGCTCTCAAACAGTTAGTCGTAGCTGGTAACGTCCTGCTGTACCTACCGGAACCGGAAGGGTCAAACTATAATCCCATGAAGCTGTACCGATTGTCTTCTTATGTGGTCCAACGAGACGCATTCGGCAACGTTCTGCCAATGGTGACTCGTGACCAGATAGCTTTTGGTGCTCTCCCTGAgg                             >  1:10228/2‑301 (MQ=255)`  
                                                                                                                                                                                                                                                                                `ggagatgtgtataagggacagccGATGAACTCGCTAAGGTCGATGAGGGCCTCTCGATGGTAGAGCGTATCATCATGAACTACATTGAGTCTAACAGTTACCGCGTGACTCTCTTTGAGGCTCTCAAACAGTTAGTCGTAGCTGGTAACGTCCTGCTGTACCTACCGGAACCGGAAGGGTCAAACTATAATCCCATGAAGCTGTACCGATTGTCTTCTTATGTGGTCCAACGAGACGCATTCGGCAACGTTCTGCAAATGGTGACTCGTGACCAGATAGCTTTTGGTGCTCTCCCTGAGGa                            <  1:64657/280‑1 (MQ=255)`  
                                                                                                                                                                                                                                                                                    `gcaGTTACTGAGCGACCCCGATGAACTCGCTAAGGTCGATGAGGGCCTCTCGATGGTAGAGCGTATCATCATGAACTACATTGAGTCTAACAGTTACCGCGTGACTCTCTTTGAGGCTCTCAAACAGTTAGTCGTAGCTGGTAACGTCCTGCTGTACCTACCGGAACCGGAAGGGTCAAACTATAATCCCATGAAGCTGTACCGATTGTCTTCTTATGTGGTCCAACGAGACGCATTCGGCATCGTTCTGCAAATGGTGACTCGTGACCAGATAGCTTTTGGTGCTCTCCCTGAGGACATc                        >  1:113955/1‑301 (MQ=255)`  
                                                                                                                                                                                                                                                                                    `gcaGTTACTGAGCGACCCCGATGAACTCGCTAAGGTCGATGAGGGCCTCTCGATGGTAGAGCGTATCATCATGAACTACATTGAGTCTAACAGTTACCGCGTGACTCTCTTTGAGGCTCTCAAACAGTTAGTCGTAGCTGGTAACGTCCTGCTGTACCTACCGGAACCGGAAGGGTCAAACTATAATCCCATGAAGCTGTACCGATTGTCTTCTTATGTGGTCCAACGAGACGCATTCGGCAACGTTCTGCAAATGGTGACTCGTGACCAGATAGCTTTTGGTGCTCTCCCTGAGGACATc                        >  1:237446/1‑301 (MQ=255)`  
                                                                                                                                                                                                                                                                                     `caGTTACTGAGCGACCCCGATGGACTCGCTAAGGTCGATGAGGGCCTCTCGATGGTAGAGCGTATCATCATGAAC‑ACATTGAGTCTAACAGTTACCGCGTGACTCTCTTTGAGGCTCTCAAACAGTTAGTCGTAGCTGGTAACGTCCTGCTGTACCTACCGGAACCGGAAGGGTCAAACTATAATCCCATGAAGCTGTACCGATTGTCTTCTTATGTGGTCCAACGAGACGCATTCGGCAACGTTCTGCAAATGGTGACTCGTGACCAGATAGCTTTTGGTGCTCTCCCTGAGGACATCCg                      >  1:226187/1‑301 (MQ=255)`  
                                                                                                                                                                                                                                                                                       `gTTACTGAGCGACCCCGATGAACTCGCTAAGGTCGATGAGGGCCTCTCGATGGTAGAGCGTATCATCATGAACTACATTGAGTCTAACAGTTACCGCGTGACTCTCTTTGAGGCTCTCAAACAGTTAGTCGTAGCTGATAACGTCCTGCTGTACCTACCGGAACCGGAAGGGTCAAACTATAATCCCATGAAGCTGTACCGATTGTCTTCTTATGTGGTCCAACGAGACGCATTCGGCAACGTTCTGCAAATGGTGACTCGTGACCAGATAGCTTTTGGTGCTCTcctgtctcttatacac                     >  1:148056/1‑287 (MQ=255)`  
                                                                                                                                                                                                                                                                                           `cggAGCGACCCCGATGAACTCGCGAAGGTCGATGAGGGCCTATCGATGGTAGAGCGTATAATCATGAACTACATTGAGTCTAACAGTTACCGCGTGACTCGCTTTGAGGCTCTCAAAAAGTTAGTCGTAGCTGGTAACGTCCTGCTGTACCTAACGGAACCGGAAGGGTCAAACTATAATCCCATGAAGCTGTACCGATTGTCTTCTTATGTGGTCCAACGAGACGCATTCGGCAACGTTCTGCAAATGGTGACTCGTGACCAGATAGCTTTTGGTGCTCTCCCTGAGGACATCCGTAAgg                 <  1:85318/299‑1 (MQ=255)`  
                                                                                                                                                                                                                                                                                            `ggAGCGACCCCGATGAACTCGCTAAGGTCGATGAGGGCCTCTCGATGGTAGAGCGTATCATCATGAACTACATTGAGTCTAACAGTTACCGCGTGACTCTCTTTGAGGCTCTCAAACAGTTAGTCGTAGCTGGTAACGTCCTGCTGTACCTACCGGAACCGGAAGGGTCAAACTATAATCCCATGAAGCTGTACCGATTGTCTTCTTATGTGGTCCAACGAGACGCATTCGGCAACGTTCTGCAAATGGTGACTCGTGACCAGATAGCTTTTGGTGCTCTCCCTGAGGACATCCGTAAGGc                <  1:297925/300‑1 (MQ=255)`  
                                                                                                                                                                                                                                                                                                 `gACCCCGATGAACTCGCTAAGTTCGATGAGGGCCTCTCGATGGTAGAGCGTATCATCATGAACTACATTGAGTCTAACAGTTACCGCGTGACTCTCTTTGAGGCTCTCAAACAGTTAGTCGTAGCTGGTAACGTCCTGCTGTACCTACCGGAACCGGAAGGGTCAAACTATAATCCCATGAAGCTGTACCGATTGTCTTCTTATGTGGTCCAACGAGACGCATTCGGCAACGTTCTGCAAATGGTGACTCGTGACCAGATAGCTTTTGGTGCTCTCCCTGAGGACATCCGTAAGGCTGTAg           <  1:127003/301‑1 (MQ=255)`  
                                                                                                                                                                                                                                                                                                  `aCCCCGATGGACTCGCTAAGGTCGATGAGGGCCTCTCGATGGTAGAGCGTATCATCATGAACTACATTGAGTCTAACAGTTACCGCGTGACTCTCTTTGAGGCTCTCAAACAGTTAGTCGTAGCTGGTAACGTCCTGCTGTACCTACCGGAACCGGAAGGGTCAAACTATAATCCCATGAAGCTGTACCGATTGTCTTCTTATGTGGTCCAACGAGACGCATTCGGCAACGTTCTGCAAATGGTGACTCGTGACCAGATAGCTTTTGGTGCTCTCCCTGAGGACATCCGTAAGGCTGTAGa          >  1:13886/1‑301 (MQ=255)`  
                                                                                                                                                                                                                                                                                                  `aCCCCGATGAACTCGCTAAGGTCGATGAGGGCCTCTCGATGGTAGAGCGTATCATCATGAACTACATTGAGTCTAACAGTTACCGCGTGACTCTCTTTGAGGCTCTCAAACAGTTAGTCGTAGCTGGTAACGTCCTGCTGTACCTACCGGAACCGGAAGGGTCAAACTATAATCCCATGAAGCTGTACCGATTGTCTTCTTATGTGGTCCAACGAGACGCATTCGGCAACGTTCTGCAAATGGTGACTCGTGACCAGATAGCTTTTGGTGCTCTCCCTGAGGACATCCGTAAGGCTGTAGa          <  1:170235/301‑1 (MQ=255)`  
                                                                                                                                                                                                                                                                                                  `aCCCCGATGAACTCGCTAAGGTCGATGAGGGCCTCTCGATGGTAGAGCGTATCATCATGAACTACATTGAGTCTAACAGTTACCGCGTGACTCTCTTTGAGGCTCTCAAACAGTTAGTCGTAGCTGGTAACGTCCTGCTGTACCTACCGGAACCGGAAGGGTCAAACTATAATCCCATGAAGCTGTACCGATTGTCTTCTTATGTGGTCCAACGAGACGCATTCGGCAACGTTCTGCAAATGGTGACTCGTGACCAGATAGCTTTTGGTGCTCTCCCTGAGGACATCCGTAAGGCTGTAGa          <  1:31715/301‑1 (MQ=255)`  
                                                                                                                                                                                                                                                                                                  `aCCCCGATGAACTCGCTAAGGTCGATGAGGGCCTCTCGATGGTAGAGCGTATCATCATGAACTACATTGAGTCTAACAGTTACCGCGTGACTCTCTTTGAGGCTCTCAAACAGTTAGTCGTAGCTGGTAACGTCCTGCTGTACCTACCGGAACCGGAAGGGTCAAACTATAATCCCATGAAGCTGTACCGATTGTCTTCTTATGTGGTCCAACGAGACGCATTCGGCAACGTTCTGCAAATGGTGACTCGTGACCAGATAGCTTTTGGTGCTCTCCCTGAGGACATCCGTAAGGCTGTAGa          <  1:274873/301‑1 (MQ=255)`  
                                                                                                                                                                                                                                                                                                  `aCCCCGATGAACTCGCTAAGGTCGATGAGGGCCTCTCGATGGTAGAGCGTATCATCATGAACTACATTGAGTCTAACAGTTACCGCGTGACTCTCTTTGAGGCTCTCAAACAGTTAGTCGTAGCTGGTAACGTCCTGCTGTACCTACCGGAACCGGAAGGGTCAAACTATAATCCCATGAAGATGTACCGATTGTCTTCTTATGTGGTCCAACGAGACGCATTCGGCAACGTTCTGCAAATGGTGACTCGTGACCAGATAGCTTTTGGTGCTCTCCCTGAGGACATCCGTAAGGCTGTAGa          <  1:61569/301‑1 (MQ=255)`  
                                                                                                                                                                                                                                                                                                  `aCCCCGATGAACGCGCGAAGGGCGATGAGGGCCTCTCGATGGTAGAGCGTATCAGCATGAACGACATTGAGTCGAACAGTTGCCGCGTGACTCACTTTGAGGCTCTCAAACAGTTAGTCGTAGCTGGTAACGTCCTGCTGTACCTACCGGAACCGGAAGGGTCAAACTATAATCACATGAAGCTGTACCGATTGTCTTCTTATGTGGTCCAACGAGACGCATTCGGCAACGTTCTGCAAATGGTGACTCGTGACCAGATAGCTTTTGGTGCTCTCCCTGAGGACATCCGTAAGGCTGTAGa          <  1:228427/301‑1 (MQ=255)`  
                                                                                                                                                                                                                                                                                                      `cGATGGACTCGCTAAGGTCGATGAGGGCCTCTCGATGGTAGAGCGTATCATCATGAACTACATTGAGTCTAACAGTTACCGCGTGACTCTCTTTGAGGCTCTCAAACAGTTAGTCGTAGCTGGTAACGTCCTGCTGTACCTACCGGAACCGGAAGGGTCACACTATAATCCCATGAAGCTGTACCGATTGTCTTCTTATGTGGTCCAACGAGACGCATTCGGCAACGTTCTGCAAATGGTGACTCGTGACCAGATAGCTTTTGGTGCTCTCCCTGAGGACATCCGTAAGGATGTAGCAGGt      >  1:219406/1‑301 (MQ=255)`  

                                                                                                                                                                                                                                                                                                           `|`                                                                                                                                                                                                                                                                                                             
`GAGAAACGAACAGGACTTGCGGAGGATGGCGCAAAGTCTGTCTATGAGCGTTTAAAGAACGACCGTGCTCCCTATGAGACACGCGCTCAGAATTGCGCTCAATATA‑CCATCCCATCATTGTTCCCTAAGGACTCCGATAACGCCTCTACAGATTATCAAACTCCGTGGCAAGCCGTGGGCGCTCGTGGTCTGAACAATCTAGCCTCTAAGCTCATGCTGGCTCTATTCCCTATGCAGACTTGGATGCGACTTACTATATCTGAATATGAAGCAAAGCAGTTACTGAGCGACCCCGATGGACTCGCTAAGGTCGATGAGGGCCTCTCGATGGTAGAGCGTATCATCATGAACTACATTGAGTCTAACAGTTACCGCGTGACTCTCTTTGAGGCTCTCAAACAGTTAGTCGTAGCTGGTAACGTCCTGCTGTACCTACCGGAACCGGAAGGGTCAAACTATAATCCCATGAAGCTGTACCGATTGTCTTCTTATGTGGTCCAACGAGACGCATTCGGCAACGTTCTGCAAATGGTGACTCGTGACCAGATAGCTTTTGGTGCTCTCCCTGAGGACATCCGTAAGGCTGTAGAAGGTCAAG  >  NC_001604.1/20246‑20843`

  

Alignment Legend

Aligned base mismatch/match (shaded by quality score): `ATCG/ATCG` `< 3 ≤` `ATCG/ATCG` `< 11 ≤` `ATCG/ATCG` `< 23 ≤` `ATCG/ATCG` `< 34 ≤` `ATCG/ATCG` `< 38 ≤` `ATCG/ATCG`

Unaligned base: `atcg`    Masked matching base: `atcg`    Alignment gap: `‑`    Deleted base: `‑`
